# Engineered Maturation Approaches of Human Pluripotent Stem Cell-Derived Ventricular Cardiomyocytes

**DOI:** 10.3390/cells9010009

**Published:** 2019-12-18

**Authors:** Feixiang Ge, Zetian Wang, Jianzhong Jeff Xi

**Affiliations:** 1 State Key Laboratory of Natural and Biomimetic Drugs, Department of Biomedical Engineering, College of Engineering, Peking University, Beijing 100871, China; ge_fei_xiang@163.com; 2 Institute of Microelectronics, Peking University, Beijing 100871, China; zt.wang@pku.edu.cn

**Keywords:** human pluripotent stem cell, cardiomyocytes, ventricular, cardiac tissue engineering, maturation

## Abstract

Heart diseases such as myocardial infarction and myocardial ischemia are paroxysmal and fatal in clinical practice. Cardiomyocytes (CMs) differentiated from human pluripotent stem cells provide a promising approach to myocardium regeneration therapy. Identifying the maturity level of human pluripotent stem cell-derived cardiomyocytes (hPSC-CMs) is currently the main challenge for pathophysiology and therapeutics. In this review, we describe current maturity indicators for cardiac microtissue and microdevice cultivation technologies that accelerate cardiac maturation. It may provide insights into regenerative medicine, drug cardiotoxicity testing, and preclinical safety testing.

## 1. Introduction

It is well-acknowledged that significant differences in hearts exist between human and model organisms. These morphological and physiological differences can lead to complex problems, such as low pathological reproducibility in clinical practice [1]. On the other hand, human pluripotent stem cells (hPSCs), including human embryonic stem cells (hESCs) and human-induced pluripotent stem cells (hiPSCs), benefitting from the property of indefinite proliferation in vitro and the capacity to differentiate into different types of somatic cells, are a promising tool in biomedical applications. Compared with the transdifferentiation of human somatic cells, the differentiation of hPSCs seems to be more efficient in terms of productivity, safety, and cost. The rapid development of hPSC research in the past few decades has made it possible to utilize hPSC-derived cardiomyocytes in large-scale cardiac tissue engineering directly.

Several effective protocols have been successfully developed to induce hPSCs to become cardiomyocytes. Embryoid body (EB) was first used in the differentiation of cardiomyocytes from hESCs and hiPSCs; however, its effectiveness and reproducibility were found to be problematic because of serum quality instabilities and heterogeneous EB sizes [2]. Differentiation protocols developed using serum-free and compound-defined media were subsequently used and improved the efficiency and reproducibility of cardiomyocytes generated from EBs. Engineering approaches to the production of homogeneous EBs emerged several years ago [3,4]. This method produced more homogenously sized EBs compared with conventional methods that used 96-well plates, and it was also appropriate to scale-up. Because of the limitations of EB protocols, monolayer three-dimensional (3D) approaches have drawn increasing attention over the past several years. Uniform hESC colonies were plated on Matrigel via a microcontact approach and the size range was optimized for maximizing mesoderm formation and cardiac induction. The method of activation of canonical Wnt signaling by the glycogen synthase kinase-3 (GSK-3) inhibitor (CHIR99021) followed by inhibition of Wnt signaling via Inhibitor of Wnt Production 2 (IWP2) or Inhibitor of Wnt Production 4 (IWP4) were found to be sufficient to produce numerous functional cardiomyocytes from multiple human pluripotent stem cell lines in two weeks without exogenous growth factors or genetic manipulation in adherent culture or suspension culture system [5,6,7]. Paul et al. designed a cardiomyocytes differentiation strategy by using a medium including three components: RPMI-1640, L-ascorbic acid 2-phosphate, and rice-derived recombinant human albumin [8]. It is essential for cardiac development in vitro through an appropriate addition of different growth factors, including fibroblast growth factor-2 (FGF-2), transforming growth factor-β (TGF-β), superfamily growth factors activin A, bone morphogenetic protein-4 (BMP-4), vascular endothelial growth factor (VEGF), and dickkopf WNT signaling pathway inhibitor 1 (DKK-1). All of these factors were found to assist human pluripotent stem cells generate myocardial precursor cells and cardiomyocytes when added in order [9].

In clinical practice, patient-derived hiPSC-CMs are an optimal disease model for personalized medicine involving inherited cardiac diseases and stem cell therapies to repair or replace injured heart tissues. hiPSC-CMs can be used to model several heart diseases, including Duchenne muscular dystrophy [10], Leopard syndrome [11], long QT syndrome [12], Timothy syndrome [13], Fabry disease [14], Danon disease [15], and familial hypertrophic cardiomyopathy [16]. In addition, hiPSC-CMs from mitochondrial cardiomyopathy of Barth syndrome (BTHS) have been used to generate a platform for pathogenesis and medical therapeutics. This cardiomyopathy model shows irregular sarcomeres, abnormal myocardial contraction, and defective heart function; more importantly, it mimics mitochondrial functional impairment caused by a mature cardiolipin defect [17]. Masahide et al. constructed a Torsade de Pointes (TdP) arrhythmias model from hiPSCs to mimic a patient’s disease condition and provide a chance to study the mechanisms of TdP generation and develop an anti-arrhythmias drug test [18]. Overexpression of CDK1, CDK4, cyclin B1, and cyclin D1 efficiently induced cell cycle progress in at least 15% of post-mitotic murine and human cardiomyocytes [19]. Nutlin-3a can selectively activate the p53 signaling pathway and induce cell apoptosis of DNA-damaged iPSCs except for DNA-damage-free cells. These iPSC-CMs were engrafted into an ischemic mouse heart to enhance mouse cardiac beating [20]. This technology may bring about potential benefits for patients with a cardiac disease in clinical medicine.

However, evidence indicates that cardiomyocytes differentiated by these approaches are not as mature as an adult phenotype, thus they may not be able to reflect the physiological response of the adult heart accurately. In addition, with respect to cardiac tissue engineering, cardiomyocytes that more closely resemble those of the native myocardium would contribute more to myocardial repair. For example, as cardiovascular diseases predominantly occur in elderly humans, immature hPSC-CMs may cause modeling to be imprecise and futile [21]. In this review, we discuss the state of current approaches to obtaining more mature cardiomyocytes.

## 2. Characteristics of Mature and Immature Cardiomyocytes

The maturation level of human cardiomyocytes is crucial to clinical therapy. Immature cardiomyocytes fail to maintain full cardiac function and may lead to an aberrant remodeling of cardiac wall and cardiomyocyte hypertrophy because of differences in cell size, myofibrillar switch, conduction velocity, metabolism, and calcium handling between the two statuses. Table 1 briefly summarizes some typical physiological and chemical differences between mature human cardiomyocytes and immature hPSC-CMs and provides criteria for defining mature cardiomyocytes [22,23,24,25]. For a review of the details, we refer the reader to the work of Xiulang Yang et al. [23].

## 3. Approaches to Obtaining Mature Cardiomyocytes

In order to obtain more mature and functional hPSC-CMs, the provision of a similar physiological microenvironment in the process of cardiomyocyte development may be a feasible adult direction. In recent years, academics have performed various experiments to stimulate cardiomyocyte maturity, including biophysical, biochemical, electrophysiological, and mechanical experiments.

### 3.1. Biophysical and Biochemical Factors

Several practicable methods have been used to promote the maturation of cardiomyocytes, including long-term cultivation, a specified material, a three-dimensional culture, a microfluidic system, a co-culture with other cells following transplantation to model organisms, a dynamic sustainability system, drugs, and metabolic regulation.

Long-term cultivation and stiff matrix have been shown to enhance human pluripotent stem cell-derived cardiomyocyte sarcomere formation, calcium handling, and ion channel protein expression [26,27]. Collagen-coated polyacrylamide gels with an elastic moduli of 10 kPa have been shown to lead to aligned sarcomeres in comparison with a stiffer substrate [28]. Mihic et al. generated human-engineered cardiac tissues from hESC-CMs in a large gelatin. Human-engineered cardiac tissues were subjected to a cyclic stretch and their cell size increased, their Z discs were organized, and the Connexin-43 expression increased significantly [29]. Biohybrids of collagen and pristine graphene increased the metabolic activity of human pluripotent stem cell-derived cardiomyocytes and enhanced sarcomere structures [30].

Three-dimensional (3D) culture systems can mimic the native cardiomyocyte microenvironment in vivo to support the maturation of cardiomyocytes. Tulloch et al. generated 3D human-engineered cardiac tissues from hPSC-CMs in collagen that was seeded into a channel with a silicon floor plus nylon mesh anchors. After seven days, myofibril and Z-disc alignment increased [31]. Lee et al. used 3D bio-print collagen to obtain a human heart tissue model that possessed synchronized contraction and directional action potential propagation [32]. hESC-CMs in 3D patches exhibited more mature characteristics, including significantly faster conduction velocities and longer sarcomeres as compared with two-dimensional (2D) monolayers. The conduction velocities of these cardiac patches increased significantly as the purity of the cardiomyocytes increased [33]. Human cardiac muscle patches transplanted into swine were shown to prominently improve left ventricular function and myocardial stress, promote myocardial hypertrophy, and reduce myocardial apoptosis [34]. It was shown that a 3D culture suppressed smooth muscle -actin content and increased the expression of several cardiac markers [35].

Microfluidic systems can be used to study disease and organoid models. In recent years, combinations of microfluidic systems and functional human myocardium have been developed for drug cardiotoxicity testing [36]. Flow culture systems provide continuous gas and nutrient exchange to induce cardiomyocyte maturation. Dynamic cultures result in an enhancement in sarcomeric protein expression, an increase in size, augmentation of the contraction force, and a higher conduction velocity [37].

In addition, mixtures of human primary or human-induced pluripotent stem cell-derived cardiomyocytes (hiPSC-CMs), fibroblasts, and endothelial cells have been used to obtain vascularized functional myocardium. This improvement has allowed us to introduce blood flow into cardiomyocyte cultivation systems and paves the way to cardiomyocyte metabolism and maturation [38]. Human cardiomyocyte patches, through several types of cells derived from hPSCs, were shown to enhance the capacity for excitation contraction coupling, calcium handling, and force generation. Moreover, Johannes and co-workers found that co-transplantation of hESC-derived epicardial cells and cardiomyocytes could double the cardiomyocyte proliferation and augmented angiogenesis between the graft and the host simultaneously [39]. Triiodothyronine is essential to myosin heavy chains (MHCs) and Titin isoform switchover in normal cardiac development. Addition of triiodothyronine was shown to compel immature cardiomyocytes to show several maturation characteristics [40]. The co-inhibition of HIF1 (hypoxia-inducible factor 1) and lactate dehydrogenase A promoted the function maturation of hPSC-CM as mitochondria prefer to conduct oxidative phosphorylation rather than aerobic glycolysis and resulted in sarcomere length increase and contraction stress enhance [41].

These biophysical and biomedical approaches promote the growth and proliferation of immature cardiomyocytes and the formation of adult-like cardiac tissue with an organized ultrastructure, longer sarcomeres, more intensively developed mitochondria, more T-tubules, a more mature oxidative metabolism, and more rapid calcium handling.

### 3.2. Electrophysiological Stimulation

The spontaneous beating of cardiomyocytes is directly regulated by the cells of atrio-ventricular nodes in vivo. A combination of 3D cultivation with 6 Hz of electrophysiological stimulation was shown to markedly increase myofibril ultrastructural organization and cardiomyocyte size, elevate the conduction velocity, and improve both electrophysiological and calcium ion handling in hPSC-CMs [42]. Electrophysiological stimulation of 2 Hz was used to culture hESC-CMs in a 3D matrix, and significant improvements in contraction and calcium handling were obtained [43]. Chiu et al. found that an electrical field with a symmetric biphasic square and strengths of 2–5 V/cm at a frequency of 1 Hz could enhance the hallmarks of cardiomyocyte maturation in vitro [44]. Thus, the maturation process of cardiomyocytes in vitro progresses more quickly when accompanied by optimized electrophysiological stimulation.

More and more researchers are becoming aware of the fact that electrophysiological stimulation can promote the maturation of hPSC-CMs. However, it remains hard to compare the results from different assays as a universal and gold standard is currently lacking. Besides this, it is obvious that diverse electric field intensities and stimulation frequencies, hPSC cell types, and cultivation conditions can lead to differences in the maturation of cardiomyocytes. Thus, there may be benefits to establish a compatible platform to assess the maturation process, and make comparisons in different stimulation models.

### 3.3. Mechanical Stress

Functional cardiomyocytes are linked to varieties of cells and structure in vivo. These structures provide the cells with anchors to contract and contribute to physiological hypertrophy. Mechanical loading may be the efficient factor with the most potential when considering the explosion of research in this area in recent years. Mechanical stress increases cells’ size and improves the contraction force that is associated with hypertrophic growth. Jianzhong et al. devised a system for assembling muscle-powered microdevices based on precise manipulation of materials to monitor muscle tissue function [45]. Furthermore, periodic stretching of hPSC-CMs in a 3D structure mixture was shown to cause faster force production, higher calcium influxes, an increased expression of β-MHC and cTnT [35]. Schmelter et al. demonstrated that cyclic mechanical stretching activated the Reactive Oxygen Species (ROS) signaling pathway and enhanced the differentiation of ESCs into cardiomyocytes [46]. Ronaldson et al. formed cardiac grafts from early stage iPSC-derived cardiomyocytes and trained them via cyclic mechanical stress for several weeks. After one month, the grafted cardiomyocytes showed adult-like gene expression profiles, increased sarcomere length, enhanced density of mitochondria, the presence of T-tubules, metabolism switch, and functional calcium handling [47]. Table 2 summarizes some typical engineered approaches to the maturation of human and rodent cardiomyocytes.

Mechanical loading can improve the rate of maturation of hPSC-CMs and contractile properties. All these characteristics reflect the state of maturity of these cells. However, there is little published data about the real-time monitoring of cardiomyocyte development; to date, the shortage of clinical feedback has slowed its application.

## 4. Conclusions

In this review, we summarized the approaches that have been adopted to improve the maturity of hPSC-CMs under different conditions. Human embryonic cardiac development and postnatal physiological hypertrophy processes are difficult to study because of species specificity and the lack of availability of human heart tissues. Mature hPSC-CMs may reflect the pathological state in adults more accurately and serve as preferential disease models for clinical use.

With the rapid development in this multidisciplinary field, our understanding of the maturation of human cardiomyocytes has been growing in recent years. Many studies on the maturation of cardiomyocytes have been published in multiple journals in the last decade. Some methods have been applied to successfully produce adult-like cardiomyocytes with respect to the biochemical indicators; however, the remaining methods still need improvement. Current hurdles include achieving adult-like cardiomyocytes with angiogenesis and organized, mixed assemblies of multi-layer 3D heart tissues. A real-time assessment system is required to compare different approaches and obtain an optimized maturation status in hPSC-CMs. Besides this, in order to monitor feedback from cell and tissue signals, we may need an electrophysiological surveillance system that can regulate the differentiation and maturation of cardiomyocytes in real time in clinical practices. A mature and functional human cardiac model in vitro could play a role in myocardial tissue development research, cardiotoxicity drug screening, and clinical therapies.

## Figures and Tables

**Table 1:** Distinctions between mature human cardiomyocytes (CMs) and immature CMs.

		Mature CMs	Immature CMs
Structure	Structure	Rod-shaped	Round and irregular
	Alignment	Orderly	Disorderly
	Nucleation	20–30% binuclear or polynuclear	Slightly binuclear
	Beating	Quiescent	Spontaneous
	Length–width ratio	5–10:1	1–3:1
	Sarcomere banding	Z discs, I band, H band, A band, M band	Z discs, I band
	Sarcomere length	2.2 μm	1.6 μm
	Troponin	cTnT, high β-MHC/α-MHC, high MLC2v/MLC2a, high cTnI/fetal ssTnI, Titin isoform N2B, ADRA1A	cTnT, low β-MHC/α-MHC, nondeterministic MLC2v/MLC2a, low cTnI/fetal ssTnI, Titin isoform N2BA
	SRP	High CSQ, PLN, RYR2, SERCA/ATP2A2	Low CSQ, PLN, RYR2, SERCA/ATP2A2
	T-tubules	Present	Not present
	Mitochondria	Regularly distributed; 20–40% of cell volume	Irregularly distributed; paucity
	LGJ	Intercalated discs	Circumferential
Biochemistry	Metabolism	Fatty acid β-oxidative	Glycolysis and lactate
Biophysical	Force	40–80 mN/mm^2^ for muscle lines μN range for a single cell	0.08-4 mN/mm^2^ for 3D cultivation nN range for a single cell
Electrophysiology	Capacitance	150 pF	10–30 pF
	RMP	−80 to −90 mV	−20 to −60 mV
	Upstroke velocity	100–300 V/s	10–50 V/s
	Conduction velocity	60 cm/s	10–20 cm/s
	APA	100–110 mV	70–120 mV

cTnT, Cardiac troponin T2; β-MHC, Myosin heavy β chain; MLC2v, Myosin light chain 2 ventricular isoform; MLC2a, Myosin light chain 2 atrial isoform; ADRA1A, Adrenoceptor α1A; cTnI, Cardiac troponin I3; CSQ, Calsequestrin; PLN, Phospholamban; RYR2, Ryanodine receptor 2; SERCA/ATP2A2, Sarco/endoplasmic reticulum calcium transport ATPases; SRP, Sarcoplasmic Reticulum Proteins; T-tubule, Transverse tubule; RMP, Resting Membrane Potential; LGJ, Location of Gap Junctions; APA, Action Potential Amplitude.

**Table 2:** Different methods for maturing cardiomyocytes.

Stimuluses	Cultured Cell Types	Maturation Conditions	Reference
Electric stimulation	Hes3 hESCs	After 4 days of culturing in the presence of electric field stimulation (a 6.6 V/cm, 1 Hz, and 2 ms pulse), hESC-CM elongation and troponin-T enhancement.	[48]
	Hes2 and Hes3 hESCs and CDI-MRB HR-I-2Cr-2R hiPSCs	Biowires increased myofibril ultrastructural organization, elevated conduction velocity, improved Calcium handling properties, and produced better electrophysiological performance.	[42]
	C25 hiPSCs	2 Hz in the first week and 1.5 Hz thereafter, developed 1.5-fold contractile forces.	[49]
	hiPSC-CMs (ReproCardio 2)	Efficient electrical stimulations were formed by a hydrogel-based microchamber with organic electrodes. The large interfacial capacitance of the electrodes eliminated cytotoxic bubbles.	[50]
Electric stimulation and mechanical strain	Neonatal Rat Heart Cells	Engineered heart muscle was subjected to electric stimulation at 0, 2, 4, or 6 Hz for 5 days and engineered flexible poles facilitated auxotonic contractions by straining. Force–frequency relationships of 2 and 4 Hz stimulation were divergent.	[51]
	C2A, WTC-11, IMR90, and BS2 hiPSCs	After the first contraction was observed, tissue was immediately subjected to 21 days of increasingly intense electromechanical strain. Cell properties were then evaluated by a multiplex test.	[52]
Mechanical loading	HES2 hESCs	Cyclical stretching produced Cardiac troponin T elevation, cell elongation, and an increase in gap junction.	[29]
	IMR90 ESCs and IBJ hiPSCs	Compared to a 2D culture, a 3D environment increased the number of cardiomyocytes and decreased the number of smooth muscles. With cyclic stress, expression of several cardiac markers increased, including β-myosin heavy chains and cardiac troponin T.	[35]
Mechanical loading and vascular co-culture	H7 hESCs	Cyclic stress enhanced cardiomyocyte hypertrophy and proliferation rates significantly and endothelial cells showed the formation of vessel-like structures.	[31]
Textile based-culturing	UTA.04602 hiPSC	Gelatin-coated polyethylene terephthalate-based textiles were used as the culturing surface. hiPSC-CMs showed improved structural properties.	[53]
Substrate stiffness	Neonatal Rat Ventricular Myocytes	Substrates of varying elastic moduli were fabricated. Cardiomyocytes matured on 10 kPa gels were similar to the native myocardium and generated a greater mechanical force and the largest calcium transients.	[28]
